# What Doesn't Kill You Makes You Wary? Effect of Repeated Culling on the Behaviour of an Invasive Predator

**DOI:** 10.1371/journal.pone.0094248

**Published:** 2014-04-04

**Authors:** Isabelle M. Côté, Emily S. Darling, Luis Malpica-Cruz, Nicola S. Smith, Stephanie J. Green, Jocelyn Curtis-Quick, Craig Layman

**Affiliations:** 1 Earth to Oceans Research Group, Department of Biological Sciences, Simon Fraser University, Burnaby, BC, Canada; 2 Department of Marine Resources, Ministry of Agriculture, Marine Resources and Local Government, Nassau, Bahamas; 3 Cape Eleuthera Institute, Rock Sound, Eleuthera, The Bahamas; 4 Department of Applied Ecology, North Carolina State University, Raleigh, North Carolina, United States of America; Institut Pluridisciplinaire Hubert Curien, France

## Abstract

As a result of being hunted, animals often alter their behaviour in ways that make future encounters with predators less likely. When hunting is carried out for conservation, for example to control invasive species, these behavioural changes can inadvertently impede the success of future efforts. We examined the effects of repeated culling by spearing on the behaviour of invasive predatory lionfish (*Pterois volitans*/*miles*) on Bahamian coral reef patches. We compared the extent of concealment and activity levels of lionfish at dawn and midday on 16 coral reef patches off Eleuthera, The Bahamas. Eight of the patches had been subjected to regular daytime removals of lionfish by spearing for two years. We also estimated the distance at which lionfish became alert to slowly approaching divers on culled and unculled reef patches. Lionfish on culled reefs were less active and hid deeper within the reef during the day than lionfish on patches where no culling had occurred. There were no differences at dawn when removals do not take place. Lionfish on culled reefs also adopted an alert posture at a greater distance from divers than lionfish on unculled reefs. More crepuscular activity likely leads to greater encounter rates by lionfish with more native fish species because the abundance of reef fish outside of shelters typically peaks at dawn and dusk. Hiding deeper within the reef could also make remaining lionfish less likely to be encountered and more difficult to catch by spearfishers during culling efforts. Shifts in the behaviour of hunted invasive animals might be common and they have implications both for the impact of invasive species and for the design and success of invasive control programs.

## Introduction

Being hunted can change the behaviour of animals. Individuals exposed to hunting pressure spend more time being vigilant, flee from humans more readily, and are more likely to change the location or timing of their activities than individuals not exposed to hunting [1]–[3]. Such changes make survivors less likely to be encountered or captured by hunters in the future. While such behavioural responses are less studied in marine than terrestrial systems, there is evidence that active fishing can similarly alter fish behaviour [4], [5]. For example, coral reef fishes targeted by spearfishers flee sooner from a potential threat than fishes protected within no-take reserves [6], [7]. Long-term fishing pressure can also select for more passive behavioural traits, such as lower activity levels, less aggressive or bold personalities, and smaller home ranges, all of which are expected to decrease the vulnerability of surviving individuals [8], [9].

Hunting or culling is sometimes carried out for conservation purposes, such as during the eradication and control of eruptive or invasive species (e.g., [10], [11]). However, inadvertent effects of hunting on invader behaviour are often overlooked (e.g., [12]) and may impede progress towards conservation goals. For example, efforts to control non-native dingoes led to a shift in dingo activity patterns and the release of another non-native mesopredator, the feral cat, effectively causing a behaviourally mediated indirect interaction (sensu [13]) with unintentional impacts on native prey [1].

Here, we investigate the effects of control efforts on the behaviour of invasive predatory lionfish (*Pterois volitans/miles*). The rapid spread of Indo-Pacific lionfish onto coral reefs throughout the western Atlantic and Caribbean since the 1980s has generated great concern for the native reef fish and invertebrates of the region [14], [15]. The most effective and widely used strategy to reduce lionfish abundance locally is the targeted removal of individuals by spear and hand net [16], yet complete removal is rarely achieved. We currently do not know whether incomplete culling efforts affect the behaviour of remaining individuals and, subsequently, the impact of these invaders and the success of future control efforts.

To address this question, we compared the behaviour of lionfish between reefs with a known history of daytime removals of lionfish and reefs where no removals have occurred. In the native range, lionfish are crepuscular hunters [17] but daytime foraging is also commonly observed in the invaded range [18]. We hypothesized that culling would affect the activity levels, hiding patterns and flight decisions of lionfish in response to human approach. Specifically, we predicted that (1) lionfish on hunted reefs would be less active during the day, when culling usually takes place, and exhibit a more crepuscular pattern of activity than lionfish on unculled reefs, (2) lionfish would be more deeply concealed within shelters during the day than those on unculled reefs, and (3) lionfish on hunted reefs would exhibit a more wary response to humans and have a longer alert distance than lionfish on unculled reefs.

## Materials and Methods

### Ethics Statement

This study was carried out in strict accordance with the guidelines of the Canadian Council on Animal Care in Science. The protocol was approved by the Animal Care Committee of Simon Fraser University (Permit Number: 1077B-13), and covered by a research permit to the Cape Eleuthera Institute by The Bahamas Department of Marine Resources.

### Study Sites and Behavioural Data Collection

**Figure 1:**
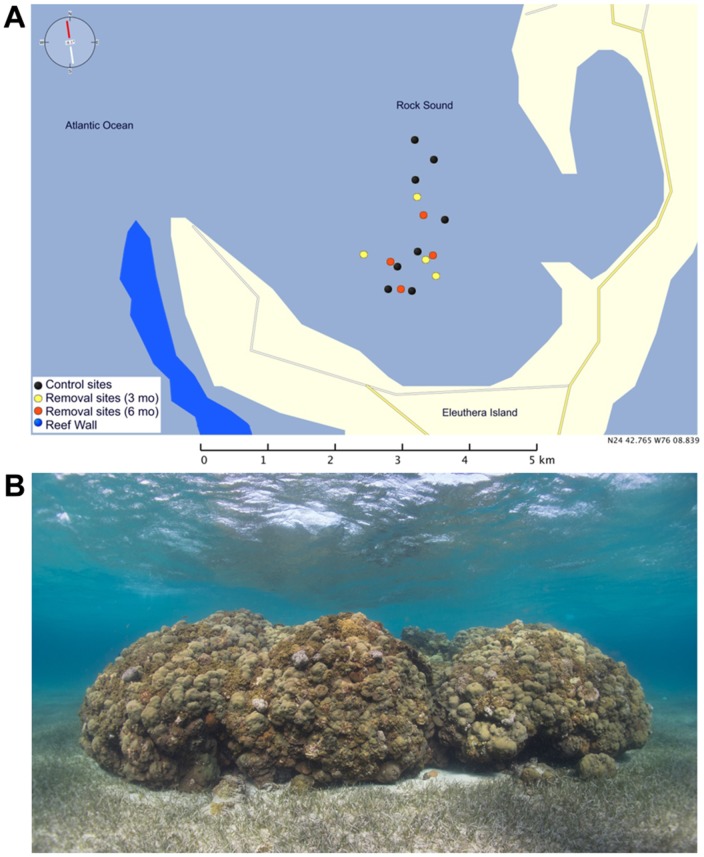
Patch reefs in Rock Sound, south Eleuthera, The Bahamas. A. Distribution of study patches in the study area. Each patch was randomly assigned on a treatment: removal of lionfish every three months (yellow dots), every six months (red dots) or no removal (black dots). The two removal regimes were combined in the analyses. B. Example of a study reef.

We conducted lionfish surveys on 16 reef patches in Rock Sound, Eleuthera Island, The Bahamas, in January 2013. Over the past two years, as part of an experiment investigating the impacts of lionfish predation, eight of these patches have been subjected to lionfish removals (every three months on four patches, every six months on four patches) by divers with non-projectile, three-prong paralyzer-tip pole spears, while no removals have occurred on the remaining eight patches. The last cull of lionfish was conducted at all eight removal sites in December 2012, three weeks prior to our study. During this cull, divers removed as many lionfish as they could, which resulted in the capture of 30-100% of lionfish initially sighted on each reef. Patches in different treatments were interspersed across the Sound (Fig. 1). All patches were at a depth of ∼ 3 m, separated by expanses of sand or seagrass, and ranged in area from 34 m^2^ to 204 m^2^. There was no difference in patch area between culled (mean ± sd, 87.8±61.5 m^2^) and unculled reefs (111.3±30.3 m^2^) (independent-samples *t*-test, *t*_10.2_ = 1.0, *P* = 0.34). The shortest distance between two adjacent patches was 300 m, which exceeds the inter-patch movement of 90% of lionfish that were tagged and resighted in our study area (N. Tamburello, unpublished data). This suggests that the patches studied were independent samples.

We used three measures to assess lionfish wariness: (1) extent of concealment at first sighting; (2) tendency towards crepuscular activity; and (3) alert distance (AD; referred to as the distance between an animal and a disturbance when the animal becomes visibly alert; sensu [19]). We assessed these measures during both daylight and crepuscular (dawn) visits to elucidate whether daily patterns of behaviour differed on culled and unculled reefs. All daytime surveys were carried out under clear sky or slightly overcast conditions to minimise the effects of cloud cover on activity patterns [18]. Water temperature, which could affect levels of lionfish activity levels, varied little during the study (mean ± sd, 22.5±1.0°C), and visibility was consistently high (∼10–15 m).

Three observers (IMC, ESD and LMC) recorded lionfish behaviour. On each patch, a pair of observers searched carefully for lionfish that were swimming, hovering above the reef or hidden in refuges. Each observer investigated the periphery of the reef first and then swam in an S-shaped pattern over the top of the reef, searching all crevices and overhangs for lionfish. Our search time was approximately 12 minutes per 100 m^2^ (range: 6 min - 19 min per reef), which is similar to the recommended searching effort for lionfish-focused surveys (15 minutes per 120 m^2^; [20]). These modified surveys yield more accurate estimates of lionfish abundance than conventional methods of underwater visual census [20], and although even lionfish-focused surveys might not detect all fish, detection probability is likely to have been similar on culled and unculled reefs. Two factors, lionfish size and reef rugosity, are known to affect the detectability of lionfish [20]. The mean length of lionfish did not differ between culled and unculled reefs (see Results). To estimate rugosity, we laid a 3 m fine-link chain to fit the contours of the reef substratum and measured the linear distance between the ends of the chain. The number of replicate measurements varied from 6 to 30, depending on reef size. Rugosity was calculated as the ratio of the chain length to the linear distance between the chain ends, with higher values denoting more rugose substrata [21]. There was no difference in rugosity between unculled (mean ± sd, 1.83±0.27, n = 8) and culled sites (1.66±0.24, n = 8; independent-samples *t*-test, *t*_13.8_ = 1.34, *P* = 0.20).

**Figure 2:**
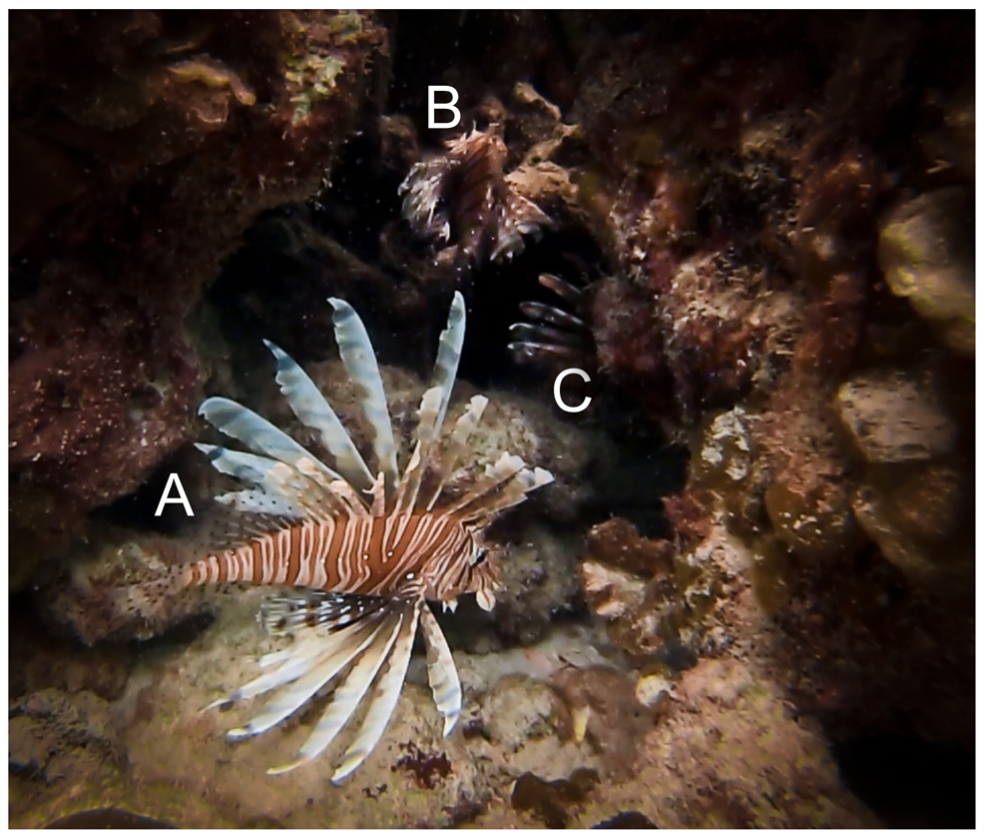
Three lionfish exhibiting a range of hiding scores. Individual A  =  hiding score 1; individual B  =  hiding score 2; individual C  =  hiding score 3. Photograph by IM Côté.

For each lionfish sighted, we estimated total length (to nearest 5 cm), categorised its behaviour at first sight as either inactive (i.e., at rest, in contact with the substrate) or active (i.e., hovering over the substrate, swimming or hunting; see [22] for description of behaviours), and assigned it a hiding score on a three-point scale (score 1: out in the open; 2: under shelter but easily visible, 3: deep into shelter and not easily visible; Fig. 2). There was substantial agreement in hiding score designation between the three observers based on a test subset of 17 lionfish not included in the study (Fleiss' Kappa = 0.68, *P*<0.001; where Kappa  =  0 indicates results that were entirely generated by chance and Kappa  =  1 indicates perfect agreement; a significant *P*-value rejects the null hypothesis of random observations; see [23]). Observers also noted the size and location of each lionfish on the reef to avoid recounting the same individual within a survey. Surveys were performed twice during daylight hours (10h00 to 15h30) and twice at dawn (06h40 to 07h30) at each site over one month, with each survey performed on a separate day, to evaluate tendency towards crepuscular activity.

On non-survey days, we visited patches during daylight to estimate alert distance (AD). The method we used was similar to that used by Gotanda et al. [4] to estimate flight initiation distance in parrotfishes. Upon locating a visible lionfish, from a consistent distance of 3–4 m, a SCUBA diver approached at the same depth as the lionfish and approximately perpendicular to the median plane of the lionfish's body, at a speed of ∼10 cm/s. At the first evidence of a response by the focal lionfish, the observer placed a weighted marker on the substrate at the level of his/her head (i.e., the nearest body part to the lionfish). The distance between the marker and the lionfish was then measured. Because lionfish are protected by venomous spines, their first reaction is not to flee immediately from an approaching diver, which most other fish species do. Instead, the behaviours denoting alertness are subtle, and included (in order of decreasing frequency): sudden flaring of dorsal and pectoral fins, orienting towards the diver with spines facing forward, or slow turning and withdrawal away from the observer. It is only upon contact with a diver (or spear) that lionfish attempt to escape with an abrupt, short swimming burst (personal observations).

### Statistical Analysis

We calculated the proportion of active lionfish, mean hiding score observed, and the proportion of high hiding scores (i.e., score  =  3) for each of the four surveys for each reef. We then averaged values for the two daytime surveys and, separately, the two crepuscular surveys conducted on each reef. Because the proportion of active lionfish and of high hiding scores can be unduly influenced by the number of lionfish present on a reef (i.e., a single active lionfish yields an estimate of 100% of fish active on a reef) and the number of lionfish per reef varied greatly (range: 0–30 individuals), we weighted these variables by the reef-specific proportion of total lionfish present across the 16 reefs, separately for day and dawn observations. Thus reefs with more lionfish contributed more to the overall means. Mean proportion of active lionfish (weighted), mean hiding scores, and mean proportion of high hiding scores (weighted) were then compared between culled and unculled patches using independent-samples *t*-tests, with reef patch as the unit of analysis. Alert distances of lionfish on culled and unculled patches were also compared using independent-samples *t*-tests, but with lionfish as the unit of analysis for two reasons: (1) it was not possible to find testable lionfish on some of the culled reefs because of low densities and inaccessibility, and (2) we had no reason to expect differences among reefs within treatment.

## Results

There were, on average, four times more lionfish on control than on culled patches, although this difference was not statistically significant (mean ± se; control: 8.1±2.9 lionfish, culled: 2.4±0.3 lionfish; *t*-test for unequal variances, *t*_7.14_ = 1.93, *p* = 0.09). We found no difference in any of the lionfish behavioural measures between four patches that were culled every 3 months and four patches that were culled every 6 months (all *t*-tests, p>0.38), thus patches culled under both regimes were combined for further analyses.

**Figure 3:**
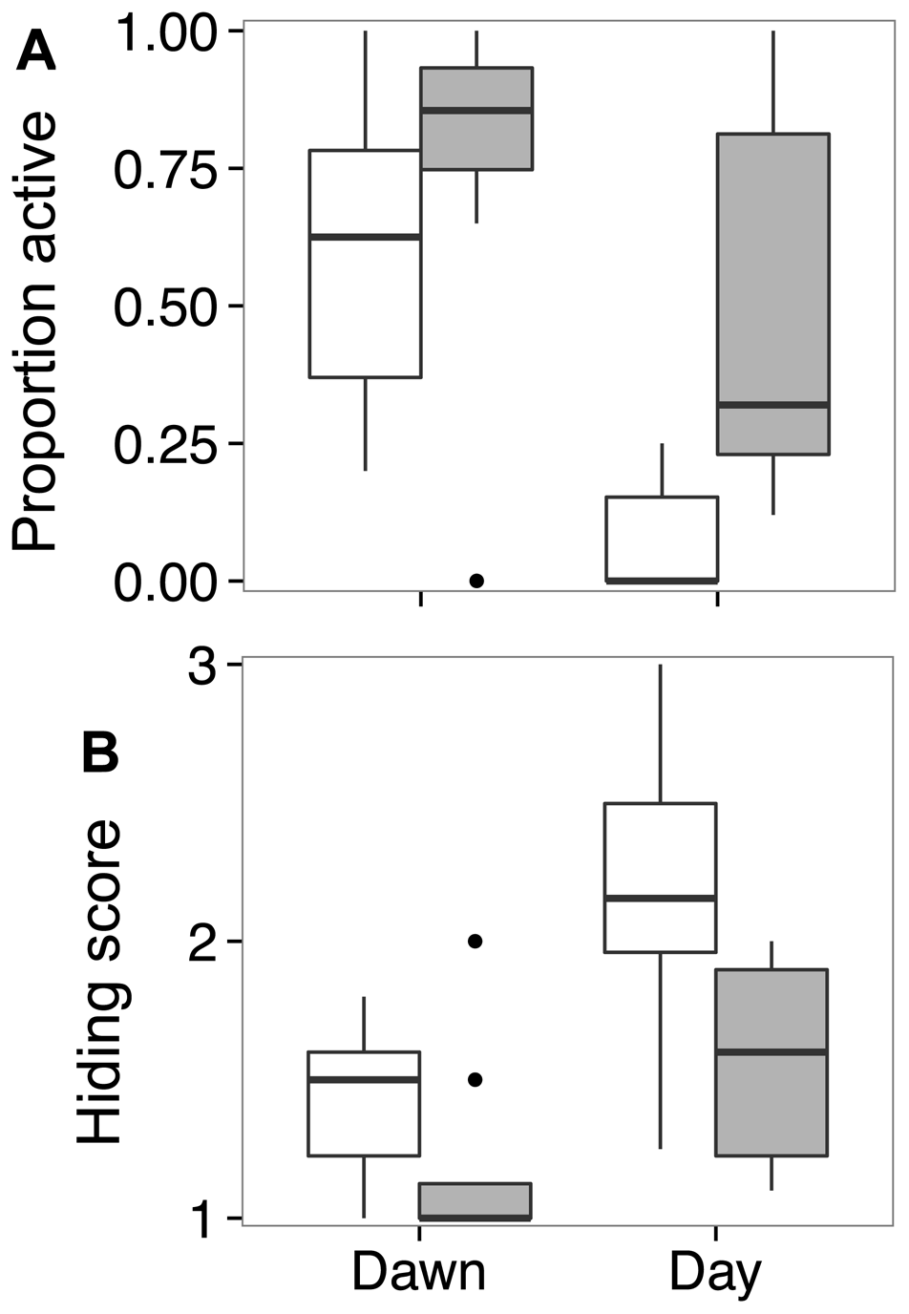
Activity patterns of invasive lionfish at dawn and midday on culled and unculled reef patches. (A) Proportion of lionfish which were active, and (B) extent of concealment, as measured on a 3-point scale, with 3 being the most hidden (see Methods; Figure 1) on culled (open bars) and unculled (shaded bars) coral reef patches in Eleuthera, The Bahamas. The proportion of active lionfish was weighted by reef-specific proportion of total lionfish present in the analysis, but it is presented unweighted in (a) for clarity. Boxplots show medians (thick horizontal lines), first and third quartiles (boxes) and 95% confidence intervals (whiskers), along with outliers (points). *n* = 8 patch reefs in all cases.

**Figure 4:**
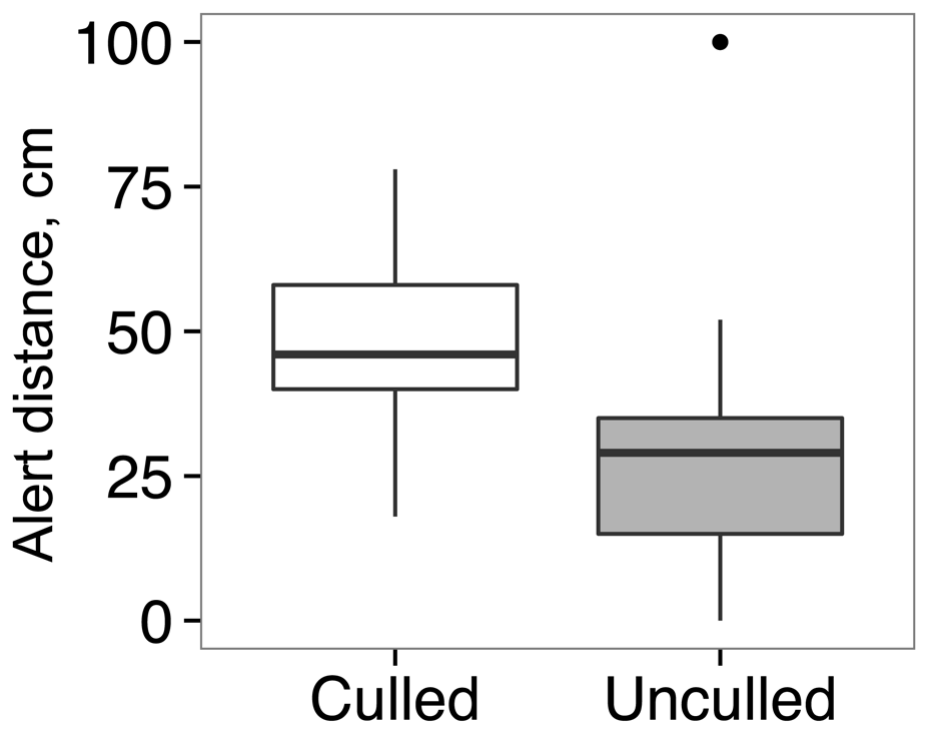
Alert distance of lionfish to an approaching diver on culled and unculled coral reef patches. Larger distances denote increased wariness. Culled patches are shown by open bars (*n* = 17 lionfish) and unculled by shaded bars (*n* = 21 lionfish). Medians (thick horizontal lines), first and third quartiles (boxes) and 95% confidence intervals (whiskers) are shown, along with an outlier (point).

During the day, a lower proportion of lionfish were active (*t*-test for unequal variances of weighted means, *t*_7.31_ = 3.07, *p* = 0.017; Fig. 3A), and lionfish were significantly more hidden (*t*-test, *t*_14_ = 2.65, *p* = 0.019; Fig. 3B), on culled than on control patches. On average, half of all lionfish on culled reefs (±45%, sd) obtained the highest hiding score, compared to 19% (±34%, sd) on unculled reefs (*t*-test on weighted means, *t*_14_ = 1.56, *p* = 0.14). However, there were no differences in activity levels or hiding scores at dawn (all tests, *p*>0.07; Fig. 3). No lionfish on any patch received a hiding score of 3 at dawn. Lionfish were generally more active and less hidden at dawn than during the day (all paired *t*-tests, *p*<0.005; Fig. 3). Lionfish were alert at a greater distance from divers on culled than on control reefs (*t*-test, *t*_36_ = 3.31, *p* = 0.002; Fig. 4). Alert distance increased weakly with lionfish total length (*r* ^2^ = 0.12, *F*_1,36_ = 4.92, *p* = 0.03), but there was no detectable difference in length between the lionfish tested on culled (25.2±1.2 cm, *n* = 17) and control reefs (22.9±1.4 cm, *n* = 21; *t*-test, *t*_36_ = 1.22, *p* = 0.23).

## Discussion

Lionfish behaved differently on coral reef patches where culling occurred than on unculled patches. On hunted patches, lionfish had depressed activity levels and were more concealed during the day when culling normally occurs. Lionfish on culled patches also reacted sooner to the presence of divers. These behavioural differences might result in lionfish being less likely encountered by spearfishers, and more difficult to capture when encountered. Our results suggest that lionfish surviving a cull could become more difficult to remove in subsequent control efforts.

There is growing evidence that active fishing, such as spearfishing, can significantly affect fish behaviour. Most of this evidence stems from comparisons between marine protected areas (MPAs), where fishing is prohibited, and adjacent fished areas. In general, fishes within MPAs have shorter flight initiation distances (FID) than those in unprotected areas, suggesting they are less wary than fishes exposed to fishing pressure [4], [6], [24]. Fishes at protected sites also have more ‘natural’ escape responses, i.e. they seek the closest shelter, whereas in hunted areas, they attempt to escape in open water [25]. A ‘fear effect’ is also detectable along gradients of fishing pressure: diver-elicited FID increases with fishing pressure in species targeted by spearfishers but not in those targeted by other methods, such as hook and line [7]. A common characteristic of these studies is the presence of continuous fishing pressure at fished sites. In contrast, fishing pressure in our study was intense, but intermittent (i.e., once every 3 to 6 months) – a situation that mirrors many lionfish control efforts occurring across the region [16]. Our results suggest that even occasional culling is enough to affect lionfish behaviour.

It is not clear whether the behavioural changes observed here, and more generally in hunted populations, are learned or evolutionary responses. The perceived threat presented by a diver may be influenced by prior experience and/or social learning [26], [27]. Thus, individuals that narrowly escape being speared or those that detect wounded conspecifics, perhaps owing to chemical alarm substances [26], might later avoid divers, which have become associated with a stressful event. Memory of stressful events, combined with high site fidelity by lionfish [28], could generate different behavioural patterns on culled and unculled patches. This learning hypothesis requires long memory retention times for lionfish, i.e. at least 3 weeks between the current study and the last culling event with no human disturbance in the intervening time, but this is well within the capability of several fish species [29]. However, the learned association should eventually attenuate, unless periodically reinforced [30]. An alternative hypothesis is that bolder, more active and/or more diurnal individual lionfish are killed in each round of culling because they are more readily targeted by spearfishers. Selective culling therefore leaves behind shier individuals with behavioural traits that make encounters with humans less likely. Such selection against individuals with bold personality traits has been documented in hunted terrestrial animals (e.g., [31]) and in fisheries using passive gear (e.g., [8], [32]).

The differences in behaviour exhibited by lionfish on hunted patches have potential ecological implications. Lionfish foraging concentrated during crepuscular periods would result in a potentially greater encounter rate with many more native fish species. The abundance of exposed reef fish typically peaks at dawn and dusk [22], [33], when there is a change-over in assemblages of nocturnal and diurnal fish species [33]–[35]. Moreover, lionfish under low-light conditions have higher hunting success than those hunting under full daylight [18], [22]. Predation rates on some native species (nocturnal, crepuscular) would therefore increase, while lionfish-induced mortality of other species (strictly diurnal) might decrease. The indirect repercussions of these changes are difficult to predict.

Our results also have implications for lionfish control efforts. Targeted removals effectively reduce lionfish densities and have demonstrated benefits for the recovery of native fish species [36]. Our results provide an explanation for the observation that complete removal of lionfish from reef patches took 30% longer to achieve than partial culling, which allowed a few lionfish to remain [36]. Our findings also suggest that in the longer term, it might become increasingly difficult and costly (in terms of person power or time) to achieve the low lionfish densities needed to mitigate the ecological impacts of this invasive predator. All else being equal, regardless of the mechanism underpinning our findings (i.e., learning or selection), lionfish on culled reefs should become more difficult to find and kill in subsequent removal efforts. This effect will arise mainly because lionfish are less active and better hidden on culled reefs, but their longer alert distance to divers on these reefs might also contribute. Although the difference in alert distances of lionfish on culled and unculled reefs was small in absolute terms (25 cm on average), it could influence spearing success. The spears used for lionfish control are usually non-projectile types (e.g., pole spears) that require very close distances to the target for accuracy, such that even experienced fishers can miss an alert lionfish [16]. The closer a diver can get to an unsuspecting lionfish, the higher the likelihood of spearing success (personal observations).

Identifying whether learning or selection explains the differences documented here is important because different mechanisms have different management consequences. If the behavioural differences stem from learning, a key question is how long-lasting these effects are. Control efforts will remain cost-effective only if culling frequency exceeds the memory retention time of lionfish, but long culling intervals might jeopardise the ecological effectiveness of these efforts in terms of recovering native prey populations [36]. If the behavioural differences stem from selection for shyness, deliberate targeting of well-hidden lionfish during culls might be required to reverse the pattern, but this would not be pragmatic in the field. Note that selection for shy lionfish could have population repercussions that favour conservation efforts, because bold personality is often positively correlated with fitness-related traits, such as foraging ability, growth and productivity [32]. A pivotal question in this case is whether the scale of current local removal efforts is sufficient to trigger such population effects in lionfish. Further study is needed to explore the likelihood of the two potential underpinning mechanisms. For example, a decline in boldness of lionfish over time, even on unculled reefs, would provide evidence of behavioural selection through culling. At present, selection for shyness seems more unlikely than the alternative, given that substantial numbers of lionfish below recreational diving depths (>30 m; Lesser and Slattery 2011; SJG, personal observations) are not exposed to culling, and the scale of current control efforts remains geographically limited. Nevertheless, whichever the mechanism at work, one thing is clear: invasive lionfish on reefs that are repeatedly culled behave differently, and these behavioural differences are likely to influence the success of current and future conservation efforts.
